# Lobular breast cancer series: imaging

**DOI:** 10.1186/s13058-015-0605-0

**Published:** 2015-07-11

**Authors:** Karen Johnson, Deba Sarma, E Shelley Hwang

**Affiliations:** Duke Cancer Institute, Duke University Medical Center, 20 Medicine Circle, Durham, NC 27710 USA

## Abstract

The limitations of mammography in the detection and evaluation of invasive lobular carcinoma (ILC) have long been recognized, presenting real clinical challenges in treatment planning for these tumors. However, advances in mammography, ultrasound, and magnetic resonance imaging present opportunities to improve the diagnosis and preoperative assessment of ILC. The evidence supporting the performance of each imaging modality will be reviewed, specifically as it relates to the pathology of ILC and its subtypes. Further, we will discuss emerging technologies that may be employed to enhance the detection rate and ultimately result in more effective screening and staging of ILC.

## Introduction

Invasive lobular carcinomas (ILCs) are the second most prevalent subtype of invasive breast cancer after invasive ductal cancer (IDC), accounting for 5 to 15 % of new breast cancer diagnoses [1–3]. Since the original description of ILC by Foote and Stewart in 1946 [4], there have been several histopathologic variants of ILC reported, which may account for the variability in the reported incidence of ILC across studies [3, 5]. The past two decades have seen a rise in the incidence of ILC. Although this increased rate is likely multifactorial, one of the most well-described risk factors associated with the increased detection of ILC is the use of postmenopausal hormone replacement therapy [2, 6].

The molecular and pathologic features that distinguish ILC from the more common IDC have been well described, and shed light on the clinical findings associated with lobular cancers [7]. ILC tends to grow more diffusely, with neoplastic cells invading the stroma in a single-cell fashion, often without a significant desmoplastic reaction. When compared with IDC, ILC tends to present at a larger size and later tumor stage, although it has a more favorable stage-matched outcome compared with IDC [8]. From a molecular standpoint, ILC is known to be more commonly estrogen receptor-positive and HER2-negative, and is characterized by loss of the adhesion molecule E-cadherin. The more prevalent use of E-cadherin staining in the diagnosis of ILC may have impacted the perceived increased incidence of this histologic type [7]. Compared with ductal cancers, ILC commonly presents as multifocal disease, and some series report a higher incidence of bilateral breast cancer, although this finding has not been consistently demonstrated across all studies.

The low density of tumor cells and lack of desmoplastic stromal reaction in ILC account for the difficulty in its detection on physical examination, mammography, and even gross pathologic evaluation. Mammograms have been found to have a low sensitivity (57 to 79 %) in detecting ILC, with up to 30 % of cases not visualized at all on mammography, and 35 % of lesions only visible on one view [9]. The low sensitivity of mammography has generated interest in other imaging modalities, such as ultrasound (US), magnetic resonance imaging (MRI), tomosynthesis and molecular targeted imaging for ILC. The radiographic features of ILC as well as the strengths and limitations of each modality are discussed below.

### Mammography

The ultimate goal of mammography is early detection of breast cancer. High quality, high resolution detailed images that exploit contrast differences between normal and diseased breast tissue are the fundamental elements that allow detection of malignancy on mammogram. When these contrast differences are small, the detection of breast cancer on mammography becomes increasingly challenging. In particular, the detection of ILC on mammography is notoriously difficult, largely due to the growth pattern with which this tumor infiltrates the breast tissue. This relatively uncommon tumor characteristically spreads by diffuse infiltration of single rows of malignant cells in a manner that does not destroy underlying anatomic structure or incite a substantial connective tissue reaction. Thus, in its early stages of development and even in later stages when substantial extent of disease is present, ILC can often escape detection on mammography [10].

The sensitivity of mammography for the detection of all types of invasive breast carcinomas ranges from 63 to 98 % [11, 12]. Due in part to the histopathologic features of ILC described above, the sensitivity of mammography in detecting ILC is lower, ranging between 57 and 81 % [13–15]. Furthermore, it is well documented that the degree of fibroglandular tissue density is inversely correlated with mammographic sensitivity. When breast tissue is described as heterogeneous or extremely dense, the sensitivity of mammography for the detection of invasive tumors can be as low as 30 to 48 % [16, 17]. Berg *et al*. [18] specifically examined the performance of mammography as a function of both tumor type and breast density. Mammographic sensitivity was 81 % for IDC compared with 34 % for ILC; when only those patients with dense breast tissue were considered, sensitivities decreased dramatically to 60 % and 11 %, respectively. Due to these diagnostic challenges, it is crucial for breast imaging radiologists to be aware of the atypical and subtle mammographic patterns of ILC.

**Fig. 1:**
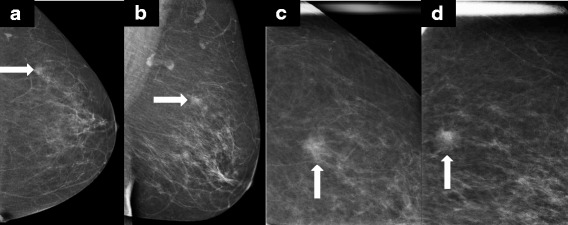
Invasive lobular carcinoma presenting as a mass on mammography. **a**,**b** Routine craniocaudal (CC) and mediolateral oblique (MLO) mammographic images detected a 1.4 cm equal density mass in the left breast. **c**,**d** Spot compression views in the CC and MLO projections better demonstrate irregular margins. Core needle biopsy was performed and revealed grade 1 invasive lobular carcinoma

A high-density spiculated mass is the hallmark mammographic manifestation of invasive carcinoma. Invasive tumors usually incite a scirrhous reaction that disrupts normal breast parenchymal architecture, resulting in spiculated margins of lesions that are readily detectable by mammography. The central high density of the mass also allows for detection based upon contrast differences between the malignant lesion and the surrounding normal breast tissue. While it has been reported in several series that up to 53 % of ILC tumors present as spiculated masses on mammography [13, 15], other investigators report that the majority of ILC tumors (68 %) present as asymmetric densities or as masses with poorly defined margins [14, 17]. All series report that a well-circumscribed mass is an uncommon mammographic presentation of ILC, seen in less than 1 % of lobular tumors. Overall, the most common mammographic manifestations of ILC include spiculated, ill-defined masses and poorly defined asymmetric densities. Both types of lesions are classically considered suspicious. Why then is the sensitivity of mammography for detecting ILC so low? The answer almost certainly is due to the lack of a conspicuous difference in density from surrounding breast parenchyma. A universally recognized confounding characteristic of these lesions described in all series is that ILC tumors lack central opacity - that is, increased density. Hilleren *et al*. [13] noted that 50 % of spiculated ILC masses have an opacity less than or equal to that of normal breast parenchyma on all views obtained. Mendelson *et al*. [19] reported that ILC tumors might even contain mammographically lucent areas. Therefore, the morphology of the ILC tumor is not so much the problem as is the lack of contrast differences between ILC tumors and surrounding, and even overlapping, normal breast tissue. This allows these tumors to be camouflaged despite being in plain view on mammogram images (Fig. 1).

Apart from spiculated, ill-defined masses and densities, the most common mammographic manifestation of ILC is architectural distortion, which accounts for approximately 14 to 25 % of cases of mammographically detected ILC [13–15]. This feature has long been part of the Breast Imaging and Reporting Data System (BI-RADS) lexicon [20] to denote an imaging finding suspicious for malignancy; however, architectural distortion can be an inconspicuous finding. Architectural distortion is identified on mammography when the normal architecture of the breast parenchyma is distorted but no discernable or discrete mass is obvious to the reader. It can include spicules radiating from a central point, as well as focal retraction or distortion of the edge of the parenchyma. Architectural distortion is the third most common mammographic manifestation of nonpalpable breast cancers and accounts for up to 45 % of missed breast cancers [21]. The subtle nature of architectural distortion is consistent with the often inconspicuous findings of ILC on mammography.

Finally, calcifications, which are readily detected on mammography, are rarely seen in ILC. The frequency of calcifications associated with ILC ranges from 1 to 25 % [14, 15, 19]. Calcifications are easily detected at mammography due to their high density, which is in noticeable contrast to background breast parenchyma. Even in extremely dense breast tissue, calcifications are typically clearly evident and prompt further investigation. The frequent absence of calcifications in ILC is an additional factor which contributes to the low sensitivity of mammography in detecting these tumors.

In summary, the mammographic appearance of ILC is often dangerously subtle. Despite the seemingly low sensitivity of mammography in detecting ILC, the ways in which these tumors manifest on mammographic images is well-documented and consistently reported. ILC most commonly presents as a spiculated, ill-defined mass/density or as architectural distortion. Both of these mammographic findings are well-established patterns known to be suspicious for invasive breast carcinoma, and breast imaging radiologists have a low threshold for performing additional imaging when these lesions are suspected in a patient with palpable findings. However, a high level of vigilance is needed to perceive more subtle features, particularly when the density of such findings is equal to or less than the background density of surrounding breast tissue. The more widespread availability of digital mammography with its superior contrast-resolution over screen film imaging has contributed to improvements in this area [22, 23]. Furthermore, some studies found digital mammography to have increased sensitivity of detecting invasive tumors compared with screen film mammography [24]. However, the direct effect of digital mammography on the detection rate of ILC is yet to be reported.

### Ultrasound

Breast US is used primarily as a diagnostic imaging tool. The most frequent indications for diagnostic breast US include interrogation of a suspected mammographic abnormality, a palpable lump, or focal breast tenderness. Originally, breast US was primarily performed to characterize a lesion as solid or cystic and to subsequently guide aspiration or biopsy. However, with improved technology and further reports elaborating its use, sonographic features are also used to distinguish benign from malignant lesions.

**Fig. 2:**
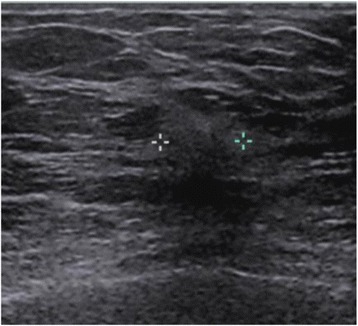
Grey-scale sonographic image of the same invasive lobular carcinoma shown in Fig. 1. Note the typical sonographic presentation with irregular margins, posterior acoustic shadowing, and disruption of normal fascial planes. Calipers are placed on the image to delineate margins of the mass

To date, there have been no screening US studies in the setting of ILC. Published studies of the sonographic appearance of ILC have all been based on diagnostic evaluations of abnormalities found at mammography or on physical exam using targeted sonography. Common physical exam findings triggering a directed breast US in these series included palpable lump, palpable thickening, palpable ipsilateral axillary lymph nodes, and nipple inversion [25, 26]. The most common sonographic appearance of ILC is a hypoechoic mass with posterior acoustic shadowing, occurring in up to 60 % of cases (Fig. 2). However, posterior acoustic shadowing may be lacking in up to 20 % of cases [25, 27]. Lobular tumors can also manifest merely as an area of posterior acoustic shadowing without an associated visibly distinct mass. In Selinko *et al*.’s series [27], 15 % of ILC tumors were described as an 'ill-defined area of altered, hypoechoic, inhomogeneous echotexture without identifiable margins and without frank shadowing', with this appearance most appreciable on extended field of view images. ILC is rarely seen sonographically as a well-circumscribed mass, reported in only 2 to 12 % of lobular tumors. Finally, as in mammography, ILC can escape detection on sonographic interrogation and over 10 % of ILC tumors are sonographically occult [25, 27].

The overall sensitivity of US for the detection of ILC is reported to be between 68 and 98 % [25–27]. It is worth noting, however, that the lower end of that range (68 %) was reported by Paramagul *et al*. [26] in the first study of its kind to report sonographic findings of ILC. At that time, the standard of care was the use of 7.5 MHz transducers, which is a much lower frequency than the 18 MHz transducers used today and in the Butler *et al*. (10 MHz) [25] and the Selinko *et al*. (13 MHz) [27] series. Advances in US technology have improved the sensitivity of sonographic detection of ILC, and this trend is only expected to continue in the future.

Comparing the sensitivities of mammography (11 to 81 %) to US (68 to 98 %) for the detection of ILC, it would appear that US is superior. However, reports focusing on the sonographic detection of ILC used US as a diagnostic tool while the investigations of mammography include both screening and diagnostic exams, precluding direct comparison between the two modalities. Nevertheless, US has been well established as an excellent tool for interrogating all palpable breast lumps, not only to distinguish solid from cystic lesions, thus helping characterize benign from malignant features, but also to exclude the presence of malignancy in the setting of negative mammogram findings. Recognizing that mammography has limited value in the detection of ILC, US plays an important role in the evaluation of suspicious physical exam findings. Butler investigated whether US could be helpful specifically in those ILC tumors that are occult on mammography. Indeed, 73 % of mammographically invisible ILC tumors were identified by directed US examination [25]. Furthermore, 92 % of 'mammographically subtle' tumors were validated by direct US scanning. These studies reinforce that in the setting of suspicious physical exam findings combined with a 'normal' mammogram, US is a very valuable adjunct to mammography in the diagnosis of ILC.

### Magnetic resonance imaging

The low sensitivity of mammography in the radiographic diagnosis of ILC has generated interest in other imaging modalities to improve early and more accurate detection. Breast MRI has an overall sensitivity of 93 % for detecting ILC, similar to the detection of breast cancers overall (90 %) [28]. However, the improved sensitivity is known to come at the cost of reduced specificity. The current uses for MRI include high-risk breast cancer screening, evaluation of equivocal mammographic and US findings, work-up for occult breast cancer in the setting of clinically positive nodes, clinically concerning nipple discharge, monitoring of chemotherapy response and, in some cases, evaluation for ipsilateral and/or contralateral breast cancers.

MRI creates images using strong magnetic fields to affect changes in the movement of protons in fat and water. The hallmark of MRI for breast imaging lies in the information gained from contrast enhancement using gadolinium. Thus, unlike mammography, which provides information on the morphology of a tumor, MRI supplies both morphologic information as well as kinetic data, thereby increasing its sensitivity in detection of malignancies. The neovascularity of malignant breast tumors typically results in rapid uptake of contrast followed by rapid washout compared with normal breast parenchyma and benign lesions. A high-field magnet strength of at least 1.5 Tesla is used to generate images with higher spatial resolution. Dedicated breast coils are now commonly employed and are designed to receive signals generated from both breast simultaneously. Images are typically acquired within 45 seconds of contrast injection and then every one to two minutes, with completion within four minutes. This enables observation of the early enhancement associated with malignant tumors.

**Fig. 3:**
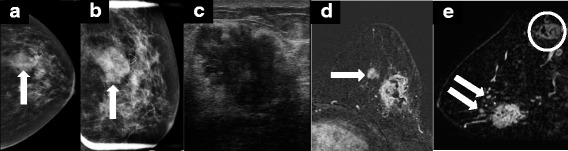
A 63-year-old female presented with a palpable mass in the left breast. **a**,**b** Mammography showed an irregular mass with partially obscured margins measuring 3.2 cm. (**c**) Directed ultrasound revealed a hypoechoic mass with irregular borders and posterior acoustic shadowing measuring 3.5 cm in the greatest dimension. **d**,**e** Core needle biopsy revealed invasive lobular carcinoma and a staging magnetic resonance image MRI was ordered (**c** and **d**). Note the irregular heterogeneously enhancing mass consistent with that seen on mammogram and ultrasound. MRI also revealed multiple smaller, enhancing masses suspicious for satellite lesions (arrows in (**d**,**e**)), as well as enlarged abnormal axillary lymphadenopathy (circle in (**e**)). Mastectomy revealed two adjacent tumors, the larger 5 cm and the smaller 3.5 cm, as well as 5 of 17 axillary nodes positive for metastatic disease

The most common MRI presentation of ILC is that of a mass with irregular or spiculated margins, followed by a non-mass lesion in 20 to 40 % of cases (Fig. 3) [28]. As expected, there is some variation in the imaging characteristics of ILC on MRI. Some studies have shown that absence of smooth margins is a typical feature of ILC; however, other reports have described ILC with smooth margins. The distribution of non-mass-like enhancement on MRI is similarly variable, and ILC may present as ductal, segmental, regional or diffuse patterns [28].

The kinetic features of ILC in comparison to IDC have also been described. As indicated previously, the typical pattern of contrast enhancement for breast cancer is rapid uptake and washout of contrast, which is usually accompanied by high peak enhancement in comparison to surrounding breast tissue. Mann *et al*. [28] compared the enhancement kinetics between ILC and IDC and found that maximum enhancement of ILC is attained at a slower rate than in IDC, but that peak enhancement is independent of tumor histology. Furthermore, a smaller percentage of ILC tumors showed delayed-phase washout in comparison to IDC.

MRI has demonstrated value in specific circumstances, such as screening for women with a >20 % lifetime predisposition for developing breast cancer [29]. However, controversies persist regarding the utility of MRI for staging and surgical planning of known breast cancers, including ILC. Studies have repeatedly shown that MRI is superior to conventional imaging, not only in terms of its increased sensitivity for detecting ILC, but also for the detection of ipsilateral and contralateral disease [30–32]. One would naturally speculate that the higher sensitivity of MRI, especially for the detection of other lesions, should improve surgical outcomes, decrease rates of recurrence, and improve overall disease-free survival. Interestingly, however, this has not been consistently proven to be the case.

In a meta-analysis of studies of MRI use in women with ILC, MRI detected additional ipsilateral disease in 32 % of cases, and 7 % of patients were found to have contralateral disease [28]. As a consequence, MRI was found to change surgical management in 28 % of cases, of which 88 % were deemed to be correct based on final pathology. In a retrospective study by the same group [33], the authors assessed the impact of preoperative MRI on the re-excision rate in ILC and found that patients who had an MRI had significantly lower re-excision rates compared with patients without preoperative MRI (9 % versus 27 %, respectively). They also concluded that there was a trend towards a lower rate of final mastectomy in the ILC subgroup, although this finding did not attain significance. A more recent meta-analysis assessing preoperative MRI use in elderly patients with IDC compared with ILC also showed that having an MRI lowered the odds of having a reoperation among women with ILC. Similar to other studies, the authors concluded that patients with ILC had a higher likelihood of undergoing a mastectomy compared with those with IDC; however, having a MRI was not significantly associated with a final mastectomy in these patients [34]. Thus, the decision to undergo a mastectomy was based on other factors, including tumor stage and biology, patient preference and surgeon bias. In contrast, however, another recent meta-analysis showed that the overall increase in mastectomies in the ILC subset of patients was in part due to the use of preoperative MRI. They also concluded that the rate of re-excision in the ILC subset receiving preoperative MRI more likely reflected the initial increase in mastectomies in these patients. Nonetheless, the authors agree that there is weak evidence to support the argument that MRI reduces the re-excision rate of ILC [35].

Despite an increase in the detection of additional ipsilateral and contralateral disease, and in some cases an ultimate change in surgical management, there is no evidence to support an overall reduction in recurrence or disease-free survival with the use of preoperative MRI. This may be due to the fact that improvement in adjuvant therapy is likely already addressing these subclinical cancers, as evidenced by the overall reduction in contralateral disease. ILCs are predominantly estrogen receptor-positive, and the 10-year rate of contralateral disease for women with estrogen receptor-positive cancer is already relatively low at 2 to 3 % [36]. The same holds true for the rate of ipsilateral breast recurrence of estrogen-positive cancers, with 10-year recurrence rate also low at 2 to 3 % [35, 37].

Detection of additional mammographically occult ipsilateral and contralateral disease may, in fact, further complicate management of the index cancer. As discussed previously, one of the limitations of MRI is its low specificity. Thus, discriminating between malignant and benign lesions in the setting of an already diagnosed ILC is likely to increase the number of biopsies and elevate patient anxiety. The need for additional biopsies can also delay treatment of the index case [38, 39]. Finally, although there is some evidence to support lower excision rates with preoperative MRI, this may come at the price of higher overall mastectomy rates.

The current evidence suggests that there could be advantages of MRI compared with conventional imaging for ILC. It is important, however, to realize that most studies looking at the role of MRI in surgical planning and outcomes, including those for ILC, are retrospective, with most studies not accounting for patient preference, tumor biology or other factors that may have influenced surgical decision-making. Despite the limitations of breast MRI, its increased sensitivity for ILC, improvement in detection of ipsilateral and contralateral lesions and possible reduction of re-excision rates makes it an important tool in combination with mammography and US in the preoperative assessment of ILC. Thus, although routine preoperative MRI for all breast cancers is not recommended, it should be considered in the setting of newly diagnosed ILC in order to better define the extent of disease.

### Emerging technologies

Advances in mammographic and sonographic technologies, such as the conversion from film-screen to digital mammography and the use of higher frequency US transducers, have provided opportunities to improve breast cancer detection rates. Significant improvements in the setting of less dense tumors such as ILC have been hampered by limitations in contrast resolution inherent to these techniques. However, several newer technologies are emerging as promising tools to add to the armamentarium of breast imaging and hold promise in detection of ILC.

**Fig. 4:**
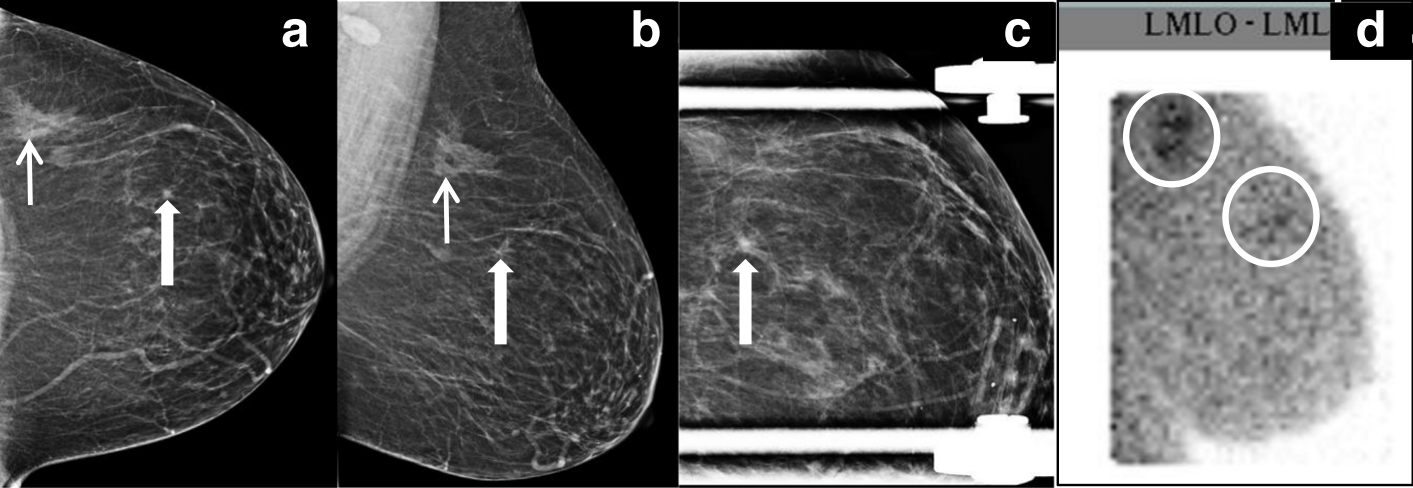
Invasive lobular carcinoma detected with molecular breast imaging. **a**,**b** Routine craniocaudal (**a**) and mediolateral oblique (**b**) views of the left breast show a focal asymmetry posteriorly (small arrow) and a small spiculated mass anteriorly (large arrow). **c** Spot compression view shows persistence of the spiculated mass. Both areas underwent core needle biopsy and revealed invasive lobular carcinoma. **d** A pre-operative staging molecular breast imaging study was performed, readily showing increased radiotracer uptake in both areas. LMLO-ML, left mediolateral oblique-mediolateral (view)

Nuclear medicine-based breast imaging was first recognized as a plausible tool to detect cancer in the 1990s. It was incidentally noted that technetium-99m-sestamibi was taken up by breast cancer in patients undergoing cardiac perfusion imaging. Distinct from traditional imaging tools that assess breast morphology and anatomy, nuclear medicine-based breast imaging is a functional study and independent of breast density. Poor spatial resolution limited early routine use of these tools; however, advances in detector technology now allow small field-of-view imaging of the breast, which produces images similar in orientation to a standard two-view mammogram with improved spatial resolution (Fig. 4). These newer generation dual-headed dedicated breast systems and the images they acquire are referred to as molecular breast imaging (MBI).

The body of literature reporting the outcomes of MBI continues to grow, elucidating its potential use as both a screening and diagnostic tool for breast cancer. In a cohort of 650 high risk, asymptomatic women presenting for screening mammography, the cancer detection rate of MBI was twice that of mammography [40]. In this series, the overall sensitivity of MBI for the detection of breast cancer was reportedly 89 % and the sensitivity of detecting ILC specifically was 79 %. Another study specifically compared the sensitivity of MBI in the detection of ILC with that of mammography, sonography, and MRI [41]. That study concluded that MBI has the highest sensitivity among the imaging modalities for the detection of ILC at 93 %. Higher radiation doses and relatively long acquisition times compared with mammography have been clear concerns related to this technology, and further research is ongoing to address these issues. Beyond these limitations, however, MBI may have a promising role as an adjunct to mammography and specifically in the detection and diagnosis of ILC, given that high sensitivities are independent of breast density and do not rely on contrast-differences to highlight abnormal findings.

Another exciting imaging tool rapidly gaining popularity for the detection of breast cancer is tomosynthesis. Breast tomosynthesis is a digital mammogram-based system that produces a series of low-dose acquisitions that are taken as an X-ray source moves in an arc over the breast. The acquisitions are then reconstructed into a series of thin slices with the intended consequence of decreasing the degree to which overlapping structures may obscure abnormal findings on traditional two-dimensional mammography. Early in the study of tomosynthesis, it was recognized that tomosynthesis has a unique strength in detecting architectural distortion, a typically subtle mammographic manifestation of malignancy [42]. Since ILC commonly presents as architectural distortion on mammography, it is reasonable to hypothesize that tomosynthesis may have an advantage over traditional two-dimensional digital mammography in the diagnosis of lobular tumors. The results of a large multi-center trial comparing cancer detection rates of digital mammography alone with digital mammography combined with tomosynthesis were reported earlier this year [43]. Digital mammography plus tomosynthesis identified more invasive breast cancers than mammography alone, representing an overall increase in cancer detection of 1.2 per 1,000. The study also analyzed their findings by histological subtype. When tomosynthesis was added to digital mammography, the detection rate for ILC increased from 0.27 to 0.55 per 1,000 cases, establishing that tomosynthesis may have a unique role in the identification of ILC. Like traditional two-dimensional mammography, tomosynthesis relies on exploiting contrast differences between normal and abnormal tissue to identify malignant lesions. However, by reducing the degree of tissue overlap and superimposition of structures, slight differences in contrast and subtle morphologic disruption of tissue due to malignancy are more conspicuous and result in improved detection of all invasive breast cancers, and may prove particularly useful for detecting ILC.

Clearly, there is much interest in evaluating novel techniques given the limitations of current imaging tools. These include the use of optical imaging of the breast and contrast-enhanced mammography to enhance breast cancer detection. However, data to define the clinical utility of such technologies are only beginning to emerge, and the potential role of these modalities is presently not well-defined, especially in the setting of invasive lobular cancer. Ongoing investigation of these and other technologies is anticipated.

## Conclusion

Lobular cancers continue to pose a specific challenge for radiographic detection. The previous discussion has highlighted that those imaging modalities reliant on contrast resolution are particularly limited by the less cohesive growth pattern of ILC. Thus, mammography and US imaging have a lower ability to discern ILC from the background density of normal breast parenchyma. Evidence evaluating the value of MRI for detection and diagnosis of ILC are emerging and provide support that MRI may be of increased utility compared with standard mammography or US. Newer technologies such as tomosynthesis and MBI are in active development and may be useful adjuncts to mammography and US, particularly for future surgical treatment planning. Together with a clinical commitment to maintain a high level of vigilance in patients presenting with nonspecific findings, continued advances in imaging will improve the ability to provide the best outcomes for women who present with lobular cancers.

### Note

This article is part of a series on *Lobular breast cancer*, edited by Ulrich Lehmann. Other articles in this series can be found at http://breast-cancer-research.com/series/LBC

## Abbreviations

IDC: invasive ductal cancer
ILC: invasive lobular carcinoma
MBI: molecular breast imaging
MRI: magnetic resonance imaging
US: ultrasound
